# Breast and cervical cancer screening practices in nine countries of Eastern Europe and Central Asia: A population-based survey

**DOI:** 10.1016/j.jcpo.2023.100436

**Published:** 2023-12

**Authors:** Ariana Znaor, Anton Ryzhov, María Lasierra Losada, Andre Carvalho, Vitaly Smelov, Anton Barchuk, Mikhail Valkov, Elena Ten, Diana Andreasyan, Saba Zhizhilashvili, Zaure Dushimova, Lilia D. Zhuikova, Alla Egorova, Alesya Yaumenenka, Sayde Djanklich, Orest Tril, Freddie Bray, Marilys Corbex

**Affiliations:** a International Agency for Research on Cancer, Lyon, France; b Taras Shevchenko National University of Kyiv, Ukraine; c World Health Organization Regional Office for Europe, Copenhagen, Denmark; d Tampere University, Faculty of Social Sciences/Health Sciences, Tampere, Finland; e Arkhangelsk Regional Oncology Centre, Northern State University, Arkhangelsk, Russian Federation; f Scientific and Production Centre for Preventive medicine of the Ministry of Health, Bishkek, Kyrgyzstan; g International Higher School of Medicine, IUK Academic Consortium, Bishkek, Kyrgyzstan; h National Institute of Health, Ministry of Health, Yerevan, Armenia; i Tbilisi State Medical University (TSMU), Tbilisi, Georgia; j Kazakh Institute of Oncology and Radiology, Almaty, Kazakhstan; k Tomsk Regional Oncology Centre, Russian Federation; l Samara Regional Oncology Centre, Russian Federation; m N.N. Alexandrov National Cancer Centre of Belarus, Minsk, Belarus; n National Cancer Center of Uzbekistan, Tashkent, Uzbekistan; °o Cancer Regional Treatment and Diagnostics Centre, Lviv, Ukraine

**Keywords:** Breast cancer, Cervix cancer, Screening, Eastern Europe, Central Asia

## Abstract

**Background:**

Eastern Europe and Central Asia (EECA) countries have higher cervical and breast cancer mortality rates and later stage at diagnosis compared with the rest of WHO European Region. The aim was to explore current early detection practices including “dispensarization” for breast and cervix cancer in the region.

**Methods:**

A questionnaire survey on early detection practices for breast and cervix cancer was sent to collaborators in 11 countries, differentiating services in the primary health setting, and population-based programs. Responses were received from Armenia, Belarus, Georgia, Kazakhstan, Kyrgyzstan, the Russian Federation (Arkhangelsk, Samara and Tomsk regions), Tajikistan, Ukraine, and Uzbekistan.

**Results:**

All countries but Georgia, Kyrgyzstan, and the Russian Federation had opportunistic screening by clinical breast exam within “dispensarization” program. Mammography screening programs, commonly starting from age 40, were introduced or piloted in eight of nine countries, organized at national oncology or screening centres in Armenia, Belarus and Georgia, and within primary care in others. Six countries had “dispensarization” program for cervix cancer, mostly starting from the age 18, with smears stained either by Romanowsky-Giemsa alone (Belarus, Tajikistan and Ukraine), or alternating with Papanicolaou (Kazakhstan and the Russian Federation). In parallel, screening programs using Papanicolaou or HPV test were introduced in seven countries and organized within primary care.

**Conclusion:**

Our study documents that parallel screening systems for both breast and cervix cancers, as well as departures from evidence-based practices are widespread across the EECA. Within the framework of the WHO Initiatives, existing opportunistic screening should be replaced by population-based programs that include quality assurance and control.

## Highlights

• Opportunistic screening by clinical breast exam took place in most countries.
• Mammography screening mostly started from age 40, without central organization.
• Romanowsky-Giemsa staining for cervical smears remains in use in five countries.
• Concurrently, most countries introduced Pap- or HPV-based screening programs.
• Opportunistic screening with non-standard practices remains common in the region.

## 1 Introduction

In 2020, in the WHO European Region there were 576,000 estimated new cases of breast and 67,000 cases of cervical cancer [1]. Both cancers are amenable to early detection for which there is an extensive evidence base [2], now with more detailed guidelines within the corresponding WHO Initiatives launched in November 2020 [3] and March 2021 [4].

Within the WHO European Region, Eastern Europe and Central Asia (EECA) countries that are not included in the EU (Armenia, Azerbaijan, Belarus, Georgia, Kazakhstan, Kyrgyzstan, Republic of Moldova, the Russian Federation, Tajikistan, Turkmenistan, Ukraine, and Uzbekistan) have comparatively high cervical cancer incidence and mortality, as well as high breast cancer mortality [1], [5]. Breast cancer incidence in the above countries is generally lower than in EU countries, with estimated age-standardised rates (ASR, World Standard) ranging from 19.5 per 100,000 (Tajikistan) to 57.5 per 100,000 (Georgia), compared to 82.8/100,000 in EU-27, while mortality is similar or even higher (from 8.0 in Tajikistan to 23.5 in Georgia per 100,000) compared to 14.8/100,000 in the EU-27. For cervical cancer, EECA countries have high incidence rates (ASR > 14.0/100,000 in Kyrgyzstan, Kazakhstan, Republic of Moldova the Russian Federation, Turkmenistan and Ukraine), compared with 9.1/100,000 in EU-27 as well as high mortality rates (ASR > 7.0/100,000 in Kazakhstan, Republic of Moldova, Turkmenistan, Kyrgyzstan) compared with 2.9/100,000 for the EU-27 [1], [6].

Our recent analysis of breast and cervical cancers stage distributions in 10 EECA countries [5] showed later stages at presentation compared to countries in Northern and Western Europe. For example, breast cancer was most commonly diagnosed at TNM stage II, which contrasts with data available from Norway, the United Kingdom, Czechia, and Belgium, where stage I was most commonly reported [7], [8], [9], [10]. For cervix cancer, the proportion of late stage (III–IV) diagnosis was high, particularly in Republic of Moldova and Armenia where it exceeded 50%, compared with corresponding proportions of 20·3% in Norway, 21% in Northern Ireland (UK), and 36·6% in Czechia [9], [11], [12].

These countries are distinct from other countries in the WHO European Region given a shared history of centralized systems of health care, based on the Semashko model inherited from the Soviet Union and characterized by dispensarization – a set of preventive activities, including health check-ups and screening provided free of cost for population of defined age-groups mostly at primary healthcare centres – “polyclinics”. Larger (urban) polyclinics employ both general practitioners and out-patient specialists (e.g. primary care gynaecologists) [13], [14], [15], [16]. The preventive exams provided comprise annual opportunistic cervical smears starting from age 18, and commonly stained by Romanowsky-Giemsa staining, as well as clinical breast exam (CBE) for women of a broad age range [17], [18]. Target population can either self-refer, be referred by their general practitioner or recommended to participate by their employer [13], [14], [16].

In spite of extensive evidence and corresponding guidelines on prioritizing population-based and de-implementing opportunistic screening programs [19], dispensarization, including opportunistic annual screening for breast and cervix cancer across broad age groups remains common in this region [14], [17], [20].

We aimed to explore the current early detection practices in EECA countries, in particular related to opportunistic screening in the primary care setting versus the implementation of population-based program, and the modalities of for cervical cancer screening, including the use of cytology with Papanicolaou or Romanowsky-Giemsa staining.

## 2 Materials and methods

We developed a questionnaire with two separate sections on breast and cervix cancer. The survey reviewed characteristics of early detection practices at the primary care level, as well as population-based programs. For breast cancer, we collected information on the clinical breast exam within the dispensarization (whether introduced, target age-group, interval, national vs. regional, coverage) and mammography screening programs (year introduced, target age group, interval, population-based/opportunistic, coverage (%) and responsible entity. For cervix cancer, we collected information on the regular testing within dispensarization (whether introduced, target age group, interval, method, national vs. regional coverage) and screening programs (year introduced, target age-group, interval, method, population-based/opportunistic, coverage (%) and responsible entity. We defined coverage (%) as the proportion of women in the population targeted by the program who were screened in the time frame defined by the program.

All questions were open-ended apart from those defining whether there was a dispensarization or screening program in the country (yes/no) and what cervical screening test was used (Papanicolaou, Romanowsky-Giemsa, DNA-based testing for human papillomavirus -HPV DNA test, other). The English version of the questionnaire is presented in Supplementary material.

As the term “opportunistic screening” and “population-based screening” are largely absent from the terminology used in the respondent countries, we described “opportunistic” as “women have a right to a free screening exam, but they are not actively invited” and “population-based” as “women who get personal invitations from the program for which there is a central screening registry to record all screened women and generate invitations at specified intervals”, in line with WHO guidance [19].

We translated the survey into Russian and piloted it with the collaborators from the previous breast and cervical cancer collaborations on stage [5] in 2020. We modified the questions that needed clarifications and sent the final version of the survey by email in June 2021 to 13 cancer registries/cancer statistics offices in 10 countries from the above study [5]; as well as to Tajikistan’s Republic Oncologic Scientific Centre. We received replies from 11 registries in nine countries: Armenia, Belarus, Georgia, Kazakhstan, Kyrgyzstan, the Russian Federation (cancer registries of Arkhangelsk, Samara and Tomsk), Tajikistan, Ukraine, and Uzbekistan. We did not receive a reply from the Republic of Moldova, while Azerbaijan responded that the screening programs for both cancers were under development and therefore, they could not provide further information at the present time. We followed up with respondents in case their replies needed clarifications. In the results (Table 1), we present the data for Arkhangelsk, Samara and Tomsk regions of the Russian Federation combined, as the screening practices were consistent across the three regions surveyed. More details are available from the Supplementary Material: Survey respondents and the Questionnaire.

**Table 1:** Policies and practice for breast cancer screening in EECA countries, 2021.

**Country**	**Clinical breast exam within dispensarization**			**Mammography screening program**					
	**Introduced**	**Age-group**	**Interval (year)**	**Introduced**	**Age-group**	**Interval (years)**	**Type**	**Coverage** **(%)**	**Responsible organization**
Armenia	Yes	18 +	1	Yes (mid-2021)	50–69	3	Pilot	Regional	National Centre of Oncology
Belarus	Yes	18 +	1	Yes (2016)	50–69	2	Pilot	Regional	National Oncology Centre, PHC
Georgia	No	-	-	Yes (2008 Tbilisi, 2011 national)	40–70	2	Population-based	National (10–23%*)	National Screening Centre, NCDC
Kazakhstan	Yes	18 +	1	Yes (2008)	40–70	2	Population-based	National (≥ 50%)	PHC
Kyrgyzstan	No	-	-	No	-	-	-	-	-
Tajikistan	No	-	-	No	-	-	-	-	-
Ukraine	Yes	18–60	3	Yes (2017–2019)	45–65	2	Pilot	Regional	PHC
Uzbekistan	Yes	18 +	1	Yes (May 2021)	45–65	2	Pilot	Regional	PHC, MoH
Russian Federation* *	No	-	-	Yes (2013)	40–75	2	Opportunistic	National	PHC

Abbreviations: PHC – Primary Health Care (Primary health centre/dispensary or general practitioner/family doctor); NCDC – National Centre for Disease Control; MoH- Ministry of Health

* 23% for Tbilisi Municipality, 10% for other regions combined

* *Arkhangelsk, Samara and Tomsk regions

## 3 Results

**Fig. 1:**
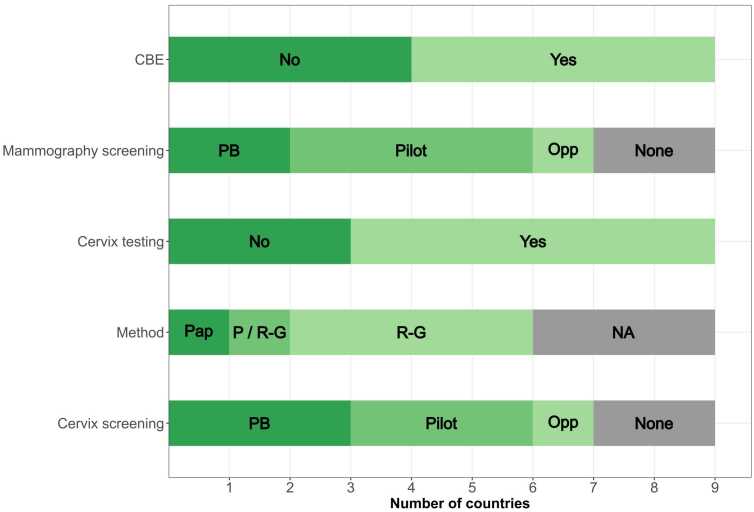
Policies and practice for breast and cervix cancer screening in EECA countries, 2021, CBE: Clinical breast exam Mammography screening: PB (population-based program), Pilot (piloting a population-based program), Opp (opportunistic screening) Cervix testing: cervical smears within dispensarization program Method: staining method used cervical smears within dispensarization program; Pap (Pap-smear), P/R-G (Pap-smear and Romanowski-Giemsa both in use), R-G (Romanowski-Giemsa) Cervix screening: PB (population-based program), Pilot (piloting a population-based program), Opp (opportunistic screening).

The screening practices for breast and cervical cancers in the respondent countries are presented in Table 1, Table 2 and Fig. 1 whereas Supplementary table 1 shows the corresponding WHO recommendations [21], [22].

**Table 2:** Policies and practice for cervix cancer screening in EECA countries, 2021.

**Country**	**Regular testing within dispensarization**				**Screening program**						
	**Introduced**	**Age-group**	**Interval (year)**	**Method (s)**	**Introduced**	**Age-group**	**Interval (years)**	**Type**	**Method (s)**	**Coverage (%)**	**Responsible organization**
Armenia	Yes	30–60	3	Papanicolau	Yes (2015)	30–60	3	Population-based	Papanicolau	National (35%, 2019–2021)	PHC
Belarus	Yes	18 +	1	R-G	No	-	-	-		-	-
Georgia	No	-	-	-	Yes (2008 Tbilisi, 2011 national)	25–60	3	Population-based	Papanicolau,	National (12–18%*)	National Screening Centre, NCDC
Kazakhstan	Yes	18 +	1	R-G	Yes (2008)	30–70	4	Population-based	Papanicolau	National (≥70%)	PHC
Kyrgyzstan	No	-	-	-	Yes (2019)	30–49	3	Pilot	VIA	Regional	PHC
Tajikistan	Yes	35–49	?	R-G	No	-	-	-	-	-	-
Ukraine	Yes	18–60	3	R-G	Yes (2017–2019)	18–65	3	Pilot	Papanicolau	Regional	PHC
Uzbekistan	No	-	-	-	Yes (2021)	30–55	7	Pilot	HPV DNA	Regional	PHC, MoH
Russian Federation* *	Yes	18 +	3	Papanicolau, R-G	Yes (2013)	18–64	3	Opportunistic	Papanicolau R-G,	National	PHC

Abbreviations: R-G – Romanowsky-Giemsa; PHC – Primary Health Care (Primary health centre/dispensary or general practitioner/family doctor); NCDC – National Centre for Disease Control; MoH- Ministry of Health, VIA -visual inspection of the cervix with acetic acid

* 18% for Tbilisi Municipality, 12% for other regions combined

* *Arkhangelsk, Samara and Tomsk regions

For breast cancer (Table 1 and Fig. 1), clinical breast exam within dispensarization remained in place in Armenia, Belarus, Kazakhstan, Ukraine and Uzbekistan, most commonly offered annually starting from the age of 18 years. All countries apart from Kyrgyzstan and Tajikistan replied that they had a mammography screening program, with the target age mostly starting from 40, with two-year intervals. Population-based mammography screening programs were introduced in Kazakhstan, while they are in a pilot phase in Armenia, Belarus and Uzbekistan. Only two respondents were able to report program coverage: Tomsk region (25.1%) and Kazakhstan (>50%). Screening programs were organized by the primary care level, apart from the National Oncology Centres in Armenia and Belarus and National Screening Centre in Georgia.

For cervix cancer (Table 2 and Fig. 1), cervical smears within dispensarization, mostly from age 18 years, remained in place in all countries but Georgia, Kyrgyzstan, and Uzbekistan. The staining method used was Papanicolaou in Armenia, Romanowsky-Giemsa in Belarus, Tajikistan and Ukraine, and a combination of the two in Kazakhstan and three regions in Russian Federation. All countries but Belarus and Tajikistan also reported having a population-based screening program as of 2021, with different target age groups, and mostly 3-year screening interval. The screening method used in population-based programs was the Papanicolaou test in most countries, while the Russian Federation reports using both officially approved Papanicolaou and, in some regions, Romanowsky-Giemsa staining. Kyrgyzstan assessed visual inspection of the cervix with acetic acid (VIA) screening in a small pilot study, whereas Uzbekistan have been piloting the use of HPV DNA testing with a 7-year interval. Population-based programs for cervix cancer were organized at the primary care level, apart from the National Screening Centre in Georgia. Three respondents were able to report the program coverage, which was low, with the exception of Kazakhstan that reported coverage of > 70%.

## 4 Discussion

To the best of our knowledge, our study is the first survey of breast and cervix cancer screening in EECA countries differentiating between dispensarization and population-based screening programs. The results of our study confirm the existence of parallel systems for early detection of breast and cervix cancer in most EECA countries, with a large volume of opportunistic screening across broad age groups taking place within the dispensarization programs, alongside population-based screening programs that are being introduced or piloted at national or regional levels. Our results provide an update of the country-specific information on mammography screening available from the WHO survey on assessing national capacity for the prevention and control of noncommunicable diseases and previous reviews [23], [24] and add novel information on screening by CBE in primary care. For cervix cancer, the latest comprehensive review of cervical cancer screening practices in the region related to the first decade of 2000 s [17]. We document that more than 10 years later, the use of Romanowsky-Giemsa staining in cervical cancer screening is still widespread across the region.

We found that for breast cancer, clinical breast examination (CBE) is offered in primary care setting to women aged 18 years and over. According to the International Agency for Research on Cancer (IARC) Handbook on breast cancer screening, there is sufficient evidence that screening by CBE alone shifts the stage distribution of tumours detected towards a lower stage, but so far inadequate evidence as to the reduction of mortality, thus CBE screening is not recommended for population-based screening [25].

In the Russian Federation, dispensarization includes mammography every two years with double reading at 40–75 years [26]. Dispensarization lacks quality control procedures, systematic follow-up after the test, and does not have individual invitations. Hence, the data are unavailable for linkages with the cancer registry, and it is impossible to calculate the program coverage. Regional differences in performance are highly likely, but the local health authorities' reports containing these data are not publicly available, and the denominators are unclear [27].

In all countries apart from Armenia and Belarus mammography screening programs started from the age 40. The working group for the IARC Handbook recommended mammography screening at ages 50–69 years [21], [25], while the latest European Guidelines on breast cancer screening published by the European Commission Initiative on Breast Cancer (ECIBC) extended the target age-group to 45–74 years [28]. Even Kazakhstan, the country with longest established national mammography screening program (since 2008), has achieved only 50% coverage for the target population of women aged 40–70. Targeting adequate coverage of narrower age group has been shown to be more efficient than expanding the age range with consequent low coverage [19]. The WHO position paper on mammography recommend screening once every 2 years for women aged 50–69, but only in countries with sufficiently robust health systems [21]. On the other hand, the early detection activities in place do seem to have some effect as according to the results of our previous study, Belarus, Kazakhstan and Ukraine have > 75% breast cancers in stages I-II, a figure over the 60% threshold specified as a goal of the WHO Breast Cancer Initiative [4].

The latest exhaustive review on cervical cancer screening in the region was published by Rogovskaya et al. in 2013,reported that cervical cancer screening in most of the Central Asian countries, the Caucasus region, the Russian Federation and the western countries of the former Soviet Union was mainly opportunistic and characterized by cytology testing, using Romanowsky-Giemsa staining [17]. More recently, several screening programs have been introduced at the national or regional level, yet parallel screening systems within dispensarization persist in seven out of nine analysed countries. The population-based programs commonly use Pap-smears, a target age commencing at either age 25 or 30, and 3-year intervals, roughly corresponding to current recommendations [22]. On the other hand, a free annual screening test, most commonly Romanowsky-Giemsa is available within dispensarization for all women aged 18 and over in most countries. While no data were collected from Turkmenistan, a recent study on cervical cancer screening in Central Asian countries confirmed the use of Romanowsky-Giemsa test in all five constituent countries [20]. Due to lack of comparative studies, and data suggesting low reproducibility and low specificity, the IARC Handbook on Cervical Cancer Screening Working Group considered this method “unclassifiable” as to its capacity to reduce the incidence of or the mortality from cervical cancer (Group C) [29].

The data from EECA countries, with rates remaining far above the elimination threshold and large proportions of cervix cancers detected in late stages [5], [30], [31], demonstrate that current practices are inadequate. The WHO guidelines for screening and treatment of cervical pre-cancer lesions recommend the use of HPV DNA detection as the primary screening test [22]. Screening women aged 30–49, either through visual inspection with acetic acid or Pap-smear every 3–5 years, or HPV DNA testing every 5 years is considered a “best buy” for cervical cancer prevention (alongside 2-dose HPV vaccination of girls aged 9–13), if linked with timely treatment of pre-cancerous lesions [2]. Modelling studies in the scope of the WHO Cervical Cancer Elimination Strategy have shown that girls-only HPV vaccination with 90% coverage alongside twice-lifetime HPV-based screening could halve cervical cancer incidence in low- and middle-income countries before 2050 [32]. At the time of our study, the only country in the region that has introduced the HPV DNA detection as the primary screening test is Uzbekistan, in a pilot launched in 2020, supported by IARC, WHO Regional Office for Europe and UNFPA [33]. HPV testing has been available as a screening option in the Russian Federation since 2020 [26]. In 2021, Belarus also introduced the legislation to start an HPV-based screening program for women 30–60 years to be tested for HPV DNA once every 5 years [34].

Breast and cervix cancer screening were included in national cancer policies and plans where available, mainly without providing details on methodology. For example, National Cancer Plan 2018–2022 for Kazakhstan has a chapter on increasing the effectiveness in cancer screening which specifies expanding screening coverage of target age groups to > 70%, as well as “the discussion of the problem of implementation of mutual responsibility for oncology screening procedures for citizens and medical organizations” [35]. Kyrgyz National Control and Prevention Strategy for Oncological Diseases, 2021–2025 specifies the introduction of pilot mammography screening for women aged 35–49, pilot screening for cervix cancer for ages 30–49 by different methods (VIA, Pap-test, HPV test) [36]. The pilot program for population-based cervix cancer screening by VIA is taking place in the Sokuluk district which comprises about 3% of Kyrgyz population. In Armenia, cervix cancer screening at ages 30–60 as well as mammography screening program pilot in regions of Lori, Tavushi and Syunik (comprising 16% Armenian population) are defined by the National Cancer Program and Action Plan [37]. In the Russian Federation, the screening recommendations are in the framework of dispensarization of adults in the Russian Federation, while the changes in methods over time were regulated by the Ministry of Health orders “on approval of the procedure for preventive medical examination and medical examination of certain groups of the adult population”, the latest of which was published in 2021 [26].

For both cancers, and in most countries in our study, screening is organized and provided at the primary care level. While the details of organization of screening programs are beyond the scope of this study, they will be further explored within the CanScreen5 project [38]. The optimal strategy for a screening program is that it is organized centrally with adequate IT systems and a dedicated team managing invitations and recalls, scheduling screening exams, and following up on further diagnostic exams and results [19]. Among seven countries with population-based screening programs, only Belarus and Georgia have a dedicated screening software. WHO Regional Office for Europe and IARC provided technical assistance for development of these software systems, within the EU funded BELMED project for Belarus [39], and with direct funding from WHO Regional Office for Europe for Georgia [18]. The introduction of a screening software in other countries would enable standardized reporting of comparable screening program indicators [38]. In our study, only a few respondents were able to report program coverage, and the figures provided should be interpreted with caution; for example, the reported high mammography coverage in Kazakhstan relates only to a selected subset of the target age-group, rather than the whole target population [18].

The strength of our study is in providing a detailed and timely review of early detection practices for breast and cervical cancers across the often overlooked EECA countries, differentiating between the dispensarization and the level of population-based programs, thus being able to identify the duplication of effort and funding, as well as inadequate screening methods in use. A limitation is that the respondents of our study were mostly affiliated to cancer registries, and not directly responsible for the screening programs. However, as these activities are commonly regulated by the same legislation and organized within the same institutions, our respondents were able, we believe, to provide adequate answers to the questionnaire.

In summary, our results indicate widespread opportunistic screening practices across the EECA countries, leading to suboptimal results. Within the framework of ongoing WHO Initiatives, current practices should be replaced by evidence-based practices within a population-based screening program that would offer greater equity and value.

## Declaration of Competing Interest

The authors declare that they have no known competing financial interests or personal relationships that could have appeared to influence the work reported in this paper.
