# Supplementary material for: Breast and cervical cancer screening practices in nine countries of Eastern Europe and Central Asia: A population-based survey
Source: J Cancer Policy. 2023 Dec;38:100436. doi: 10.1016/j.jcpo.2023.100436 (PMC10695765; doi:10.1016/j.jcpo.2023.100436)
Supplement: Supplementary file 1 — Supplementary material. [file mmc1.docx]

**Supplementary Table 1. WHO recommended methods for breast (1) and cervix (2) cancer screening**

|  | **Recommended method** | **Age-group** | **Interval** |
| --- | --- | --- | --- |
| **Cervix cancer screening** | HPV DNA testing* | 30-49 | 5-10 years |
| **Breast cancer screening**** | Mammography | 50-69 | 2 years |

*Where DNA-based testing for human papillomavirus (HPV DNA) testing is not yet operational, WHO suggests a regular screening interval of every 3 years when using visual inspection of the cervix with acetic acid (VIA) or cytology as the primary screening test

**Recommended only in countries with robust health systems

**References:**

1. World Health Organization. WHO position paper on mammography screening, <https://www.who.int/publications/i/item/who-position-paper-on-mammography-screening>; [accessed 2 May 2023].
2. World Health Organization. WHO guideline for screening and treatment of cervical pre-cancer lesions for cervical cancer prevention, second edition, <https://apps.who.int/iris/bitstream/handle/10665/342365/9789240030824-eng.pdf?sequence=1>.; 2021 [accessed 2 May 2022].
