# Supplementary material for: Breast and cervical cancer screening practices in nine countries of Eastern Europe and Central Asia: A population-based survey
Source: J Cancer Policy. 2023 Dec;38:100436. doi: 10.1016/j.jcpo.2023.100436 (PMC10695765; doi:10.1016/j.jcpo.2023.100436)
Supplement: Supplementary file 2 — Supplementary material. [file mmc2.docx]

**Supplementary Material****: Survey respondents and the Questionnaire**

In the first version of the questionnaire, we asked directly whether screening programs were opportunistic. As this term is not used in the Russian language, we have excluded that question from the current version, and added more details to the questions on program infrastructure, based on which we could differentiate between opportunistic and population-based screening.

The survey respondents were mainly affiliated to cancer registries/medical statistics offices of regional or national oncology centres. Medical statistics offices in the respondents’ countries are departments in charge of hospital statistics as well as population-based cancer registry data. As these departments are required by law to produce statistics on screen-detectable sites, the respondents were well acquainted with regulations and screening practices. In case additional input was required, the survey respondents consulted their colleagues directly involved in screening programme implementation.

**Questionnaire on screening practices:**

**Please complete for the period 2019-present (if there were changes, please use more lines):**

| **Breast cancer** | | |  | |  | |
| --- | --- | --- | --- | --- | --- | --- |
| I. Is there **dispensarization** in your country? | |  | \|  \| \| --- \|   Yes | | \|  \| \| --- \|   No | |
| If *Yes*, please complete the questions below: | | |  | |  | |
| Age-group included: | | From __________ To_____________ | Other (please specify) __________  __________________________ | | | |
| Test/Exam: | |  | | | | |
| Frequency: | | Every ____ year(s) | Other (please specify) __________ ____________________________ | | | |
| Coverage: | |  | \|  \| \| --- \|   National | | \|  \| \| --- \|   Regional | |
| If *Regional*, in which regions: | | 1. | | | | |
| (add more lines if needed) | | 2. | | | | |
|  | | 3. | | | | |
|  | | 4. | | | | |
| II. Is there a breast cancer **screening programme** in your country? | | | \|  \| \| --- \|   Yes | | \|  \| \| --- \|   No | |
| If *Yes*, please complete the questions below: | | |  | |  | |
| Is there a legislation on this program? | |  | \|  \| \| --- \|   Yes | | \|  \| \| --- \|   No | |
| If *Yes*, please list and/or enclose: | | 1. | | | | |
| (add more lines if needed) | | 2. | | | | |
|  | | 3. | | | | |
|  | | 4. | | | | |
| Coverage: | |  | \|  \| \| --- \|   National | | \|  \| \| --- \|   Regional | |
| If *Regional*, in which regions: | | 1. | | | | |
| (add more lines if needed) | | 2. | | | | |
|  | | 3. | | | | |
|  | | 4. | | | | |
| Which year did the programme start: | | From __________ To_____________ | Other (please specify) __________  __________________________ | | | |
| Implemented as: | |  | \|  \| \| --- \|   Full-scale | | \|  \| \| --- \|   Pilot | |
| Age-group included: | | From __________ To_____________ | Other (please specify) __________  __________________________ | | | |
| Test/Exam: | |  | | | | |
| Frequency: | | Every ____ year(s) | Other (please specify) __________ ____________________________ | | | |
| Is the database of target population available? | |  | \|  \| \| --- \|   Yes | | \|  \| \| --- \|   No | |
| If *Yes*, please provide more details: | \|  \| \| --- \|   up-to-date database exist at each primary health care centre | | | | | |
|  | \|  \| \| --- \|   the list of eligible women is provided by a specific institution dealing with screening (at regional or national level) | | | | | |
| Institution(s): | _____________________________________________  _____________________________________________ | | | | | |
| Does woman in the target population receive a personalised invitation by mail, email, phone or SMS ? | | | | \|  \| \| --- \|   Yes | | \|  \| \| --- \|   No |
| If *No* , please describe how are the women invited (with free text): | | ______________________________________  ______________________________________  ______________________________________ | | | | |
| Is there a screening database/registry that collects data about individual women (when they were screened, what was the result, etc): | | | | \|  \| \| --- \|   Yes | | \|  \| \| --- \|   No |
| If available, please list the population coverage: | | _______% | | | | |
| Please enclose the latest programme report available (PDF, link etc.) _____________________________  ________________________________________________________________________ | | | | | | |

| **Cervix cancer** | | | |  | |  | |
| --- | --- | --- | --- | --- | --- | --- | --- |
| I. Is there **dispensarization** in your country? | |  | | \|  \| \| --- \|   Yes | | \|  \| \| --- \|   No | |
| If *Yes*, please complete the questions below: | | | |  | |  | |
| Age-group included: | | From __________ To_____________ | | Other (please specify) _________  ___________________________ | | | |
| Test: | |  | | | | | |
| Frequency: | | Every ____ year(s) | | Other (please specify) __________ ____________________________ | | | |
| Staining method used | | \|  \| \| --- \|   Papanicolau | | \|  \| \| --- \|   Giemsa-Romanowski | | | |
| Coverage: | |  | | \|  \| \| --- \|   National | | \|  \| \| --- \|   Regional | |
| If *Regional*, in which regions: | | 1. | | | | | |
| (add more lines if needed) | | 2. | | | | | |
|  | | 3. | | | | | |
|  | | 4. | | | | | |
| II. Is there a cervix cancer **screening programme** in your country? | | | | \|  \| \| --- \|   Yes | | \|  \| \| --- \|   No | |
| If *Yes*, please complete the questions below: | | | |  | |  | |
| Is there a legislation on this program? | |  | | \|  \| \| --- \|   Yes | | \|  \| \| --- \|   No | |
| If *Yes*, please list and/or enclose: | | 1. | | | | | |
| (add more lines if needed) | | 2. | | | | | |
|  | | 3. | | | | | |
|  | | 4. | | | | | |
| Coverage: | |  | | \|  \| \| --- \|   National | | \|  \| \| --- \|   Regional | |
| If *Regional*, in which regions: | | 1. | | | | | |
| (add more lines if needed) | | 2. | | | | | |
|  | | 3. | | | | | |
|  | | 4. | | | | | |
| Which year did the programme start: | | From __________ To_____________ | | Other (please specify) _________  ____________________________ | | | |
| Implemented as: | |  | | \|  \| \| --- \|   Full-scale | | \|  \| \| --- \|   Pilot | |
| Age-group included: | | From __________ To_____________ | | Other (please specify) _________  ___________________________ | | | |
| Test: | |  | | | | | |
| Frequency: | | Every ____ year(s) | | Other (please specify) __________ ____________________________ | | | |
| Staining method used | | \|  \| \| --- \|   Papanicolau | | \|  \| \| --- \|   Giemsa-Romanowski | | | |
| Is the database of target population available? | | |  | \|  \| \| --- \|   Yes | | \|  \| \| --- \|   No | |
| If *Yes*, please provide more details: | \|  \| \| --- \|   up-to-date database exist at each primary health care centre | | | | | | |
|  | \|  \| \| --- \|   the list of eligible women is provided by a specific institution dealing with screening (at regional or national level) | | | | | | |
| Institution(s): | _____________________________________________  _____________________________________________ | | | | | | |
| Does woman in the target population receive a personalised invitation by mail, email, phone or SMS ? | | | | | \|  \| \| --- \|   Yes | | \|  \| \| --- \|   No |
| If *No* , please describe how are the women invited (with free text): | | ______________________________________  ______________________________________  ______________________________________ | | | | | |
| Is there a screening database/registry that collects data about individual women (when they were screened, what was the result, etc): | | | | | \|  \| \| --- \|   Yes | | \|  \| \| --- \|   No |
| If available, please list the population coverage: | | _______% | | | | | |
| Please enclose the latest programme report available (PDF, link etc.) _____________________________  ________________________________________________________________________ | | | | | | | |
